# Nailfold Capillaroscopy Abnormalities Correlate With Disease Activity in Adult Dermatomyositis

**DOI:** 10.3389/fmed.2021.708432

**Published:** 2021-08-10

**Authors:** Dylan Johnson, Charmaine van Eeden, Naima Moazab, Desiree Redmond, Cecile Phan, Stephanie Keeling, Robert Gniadecki, Jan Willem Cohen Tervaert, Mohammed Osman

**Affiliations:** ^1^Division of Rheumatology, Department of Medicine, Faculty of Medicine and Dentistry, University of Alberta, Edmonton, AB, Canada; ^2^Division of Neurology, Department of Medicine, Faculty of Medicine and Dentistry, University of Alberta, Edmonton, AB, Canada; ^3^Division of Dermatology, Department of Medicine, Faculty of Medicine and Dentistry, University of Alberta, Edmonton, AB, Canada

**Keywords:** dermatomyositis, nailfold capillaroscopy, capillary density, disease activity, CK

## Abstract

**Objectives:** The aim of this study was to determine the relationship between disease activity in adult patients with dermatomyositis (DM) and other biomarkers of disease activity such as C-reactive protein creatinine kinase and nailfold video capillaroscopy (NVC).

**Methods:** We performed a prospective single center study of 15 adult patients with DM. Study participants underwent two assessments at least 9 months apart including clinical, laboratory and NVC evaluations. Patients received immunosuppressive medications for their dermatomyositis, and ongoing disease activity was measured by the Myositis Intention to Treat Index (MITAX). NVC evaluation included assessment of capillary density, capillary apical diameter (mm), and the number of microhemorrhages per digit.

**Results:** Microvascular abnormalities were present in most DM patients. Of these, capillary density (4.71 vs. 6.84, *p* = 0.006) and mean apical diameter (56.09 vs. 27.79 μm, *p* = 0.003) significantly improved over the study period in concordance with improving disease control (MITAX 8.53 vs. 2.64, *p* = 0.002). Longitudinal analysis demonstrated that capillary density was independently associated with MITAX (β = −1.49 [CI −2.49, −0.33], *p* = 0.013), but not other parameters such as C-reactive protein and creatinine kinase.

**Conclusions:** Nailfold capillary density is a dynamic marker of global disease activity in adult DM. NVC may be utilized as a non-invasive point-of-care tool to monitor disease activity and inform treatment decisions in patients with DM.

## Introduction

Dermatomyositis (DM) is an idiopathic inflammatory myopathy characterized by proximal muscle weakness and characteristic cutaneous findings. The diagnosis of DM is based on clinical features, complemented by detection of myositis-specific antibodies (MSAs), elevation in muscle enzymes such as creatinine kinase (CK), muscle biopsy, and/or imaging.

Monitoring response to treatment remains a challenge as no single measure is able to capture global disease activity in DM. The International Myositis Assessment and Clinical Studies Group (IMACS) has developed a core set of disease activity and treatment response criteria for adult DM. In these criteria, monitoring response to treatment is largely clinical, with inclusion of muscle enzyme evaluation as the only biomarker. However, the relationship between disease activity and muscle enzymes is not firmly established (1) and they are therefore assigned low weighting in treatment response criteria (2). The absence of a readily available and reliable marker of disease activity therefore remains a significant gap in our ability to assess disease activity in DM.

The pathogenesis of DM is driven by small vessel vasculopathy wherein perivascular inflammation leads to a reduction in the density of capillaries, resulting in tissue ischemia and dilatation of remaining capillaries. These changes are present in skeletal muscle where they lead to muscle atrophy and weakness as well as in other areas such as the nailfolds where they may be more readily detected.

Nailfold video capillaroscopy (NVC) is a point-of-care tool for directly visualizing microvascular changes associated with connective tissue diseases (3). Detection of these abnormalities has been suggested to have a clinical role in both diagnosis and prognosis, particularly in systemic sclerosis and in patients with Raynaud's phenomenon associated with an inflammatory etiology. While microvascular changes are present in DM, the role of NVC in monitoring disease activity in adult DM has not yet been established. In this study, we performed a prospective analysis of NVC findings and disease activity in adult DM.

## Methods

### Study Population

Study participants were prospectively enrolled from the Rheumatology Clinic at the Kaye Edmonton Clinic, Edmonton, Canada. All participants met 2017 EULAR/ACR DM classification criteria (4). All patients were tested for myositis specific autoantibodies. Anti-synthetase syndrome is increasingly recognized as a unique clinical entity with rapidly progressive ILD as a predominant feature. Therefore, in order to study dermatomyositis as an isolated entity, patients with anti-synthetase antibodies were excluded from this study. Patients underwent simultaneous clinical and NVC assessments at the time of enrollment, and then again after an interval ranging from 9 to 15 months. Patients received therapy as directed by their primary DM physician. All participants enrolled provided written consent for study participation which was approved by the University of Alberta Research Ethics Office. The study design did not include patient input.

### Nailfold Video Capillaroscopy

All images were captured using a 200X Video Capillaroscope (DS Medica, Italy) by a Rheumatologist trained in NVC, as previously described (5, 6). NVC parameters were recorded as follows: mean capillary density (number of capillaries per mm averaged over 8 digits), apical capillary diameter (μm), and microhemorrhages (number of hemorrhages per digit, averaged over the 8 digits).

### Clinical Measures

The Myositis Intention to Treat Index (MITAX), as described by the International Myositis Assessment & Clinical Studies Group, is a disease activity score with seven domains: cutaneous, muscle, constitutional, skeletal, gastrointestinal, pulmonary, and cardiovascular (7). Each domain was scored from 0 to 9, and the total sum is reported giving a total score range of 0–63. The score assigned to a domain integrates both the severity of current dermatomyositis manifestations and their relative improvements or exacerbations over the proceeding 4 weeks. A score of 3 or higher for a single domain generally indicates intention to treat and indication for immunosuppression.

MITAX determination was made at the same time as NVC image acquisition by a single observer. MITAX scoring was confirmed to be consistent with their primary treating physicians' intention to treat with subsequent changes in disease management. Disease activity was considered present for a given domain for any score of one or greater. Detection of myositis specific antibodies was performed by the Mitogen Advanced Diagnostics Laboratory, Calgary, Canada. CRP and CK assays were measured by Alberta Precision Laboratories, Edmonton, Canada using Beckman Coulter DxC 800 Synchron assays. Complete blood counts, alanine aminotransferase and total bilirubin levels were measured in some patients receiving methotrexate for toxicity monitoring. As not all patients were receiving methotrexate, and these results were not routinely measured for all patient and not included in analysis of disease activity.

### Statistical Methods

Changes in clinical parameters between the two assessments was analyzed by Wilcoxon signed rank or Fisher's exact tests. The longitudinal relationship between clinical parameters was analyzed used mixed-linear model regression. A series of individual regressions were first used to examine the correlation between parameters across assessments. A combined mixed-linear model regression was then performed using NVC parameters. All analysis was performed using IBM SPSS Statistics 27.0.

## Results

### Baseline Characteristics

A total of 15 DM patient were prospectively enrolled in the study. Baseline characteristics are summarized in Table 1. Our cohort was predominantly female (93%) with a median age of 53. At the time of enrollment, patients had disease duration ranging between 0 and 6 years, with a median duration of 1 year. For six patients, their baseline assessment occurred at the time of diagnosis. The majority of patients were receiving immunosuppressive therapy, with only two patients untreated at the time of initial assessment. Baseline total MITAX ranged from 2 to 16, with a mean of 8.5, indicating that most patients had ongoing disease activity requiring immunosuppression in at least one domain. Active skin (80%), muscle (67%), skeletal (47%) and constitutional (80%) disease was common at baseline, while pulmonary (13%) and gastrointestinal (20%) involvement was less frequent.

**Table 1:** Demographic, clinical, and NVC characteristics of patients across two assessments.

	**Assessment 1**	**Assessment 2**
**Number**	15	13
**Age at baseline** (year), median (range)	52 (35–80)	–
**Duration of disease at baseline** (year), median (range)	1.0 (0–4.8)	–
**Female**, *n* (%)	14 (93)	–
**Myositis specific antibodies**, *n* (%)		
TIF1γ	7 (35)	6 (46)
Mi2	3 (20)	2 (15)
NXP2	2 (13)	2 (15)
SAE	1 (7)	0 (0)
MDA5	3 (20)	3 (23)
**Organ Involvement**, *n* (%)		
Cutaneous,	12 (80)	7 (54)^ns^
Muscular	10 (67)	3 (23)^*^
Skeletal	7 (47)	5 (8)^ns^
Pulmonary	2 (13)	1 (8)^ns^
Constitutional	12 (80)	5 (38)^ns^
Gastrointestinal	3 (20)	1 (8)^ns^
**MITAX**, mean (range)	8.5 (2–16)	2.6 (0–10)^**^
**CK** (units/L), mean (range)	233 (25–2,370)	98 (49–223)^ns^
**CRP** (mg/L), mean (range)	4.1 (0.3–21)	1.5 (0.5–4.9)^ns^
**Nailfold video capillaroscopy**		
Decreased Capillary Density, *n* (%)	12 (80)	5 (38)^*^
Mean Capillary Density (capillaries/mm), mean ± SD	4.71 ± 2.31	6.84 ± 1.29^**^
Microhemorrhages, *n* (%)	12 (80)	10 (77)^ns^
Mean Microhemorrhage (count per digit), mean ± SD	0.65 ± 0.54	0.29 ± 0.32^ns^
Mean Apical Diameter (μm), mean ± SD	56.1 ± 30.5	27.8 ± 8.5^**^
**Therapy**, *n* (%)		
Prednisone	4 (27)	0 (0)
Methotrexate	7 (47)	9 (69)
Hydroxychloroquine	6 (40)	6 (46)
Mycophenolate mofetil	3 (20)	3 (23)
Leflunomide	0 (0)	1 (08)
Toficitinib	0 (0)	1 (08)
Leflunomide	5 (33)	6 (46)
No Immunosuppression	2 (13)	0 (0)

*Statistical significance between first and second assessments as measured by Wilcoxon signed rank (means) or Fisher exact (categorical) tests; ns (p > 0.05)*,

* *(P < 0.05)*,

** *(p < 0.01). Values denoted by (-) were not re-evaluated or applicable to follow-up. MITAX (Myositis intention to treat index), CK (Creatinine Kinase), CRP (C-Reactive Protein)*.

Thirteen patients underwent a second evaluation. The follow-up interval had a mean duration of 12 months, ranging from 9 to 15 months, during which time all patients received immunosuppressive therapy as indicated in Table 1. Disease activity, as measured by MITAX, was significantly lower in follow up (8.53 vs. 2.64, *p* = 0.002). Indeed, in 12 out of 13 patients, the MITAX score decreased or remained stable. Fewer patients had active muscle disease (67% vs. 23%, *p* = 0.01). Other manifestations were also observed less frequently although they did not reach significance. The mean CK value was 233.4 at baseline and 98.5 at follow-up. The observed difference was largely driven by a single patient who had a CK of more than 2,000 at baseline and was not statistically significance (*p* = 0.72). There were no significant changes in CRP (4.05 vs. 5.89, *p* = 0.32) between assessments.

### Nailfold Video Capillaroscopy Findings

**Figure 1:**
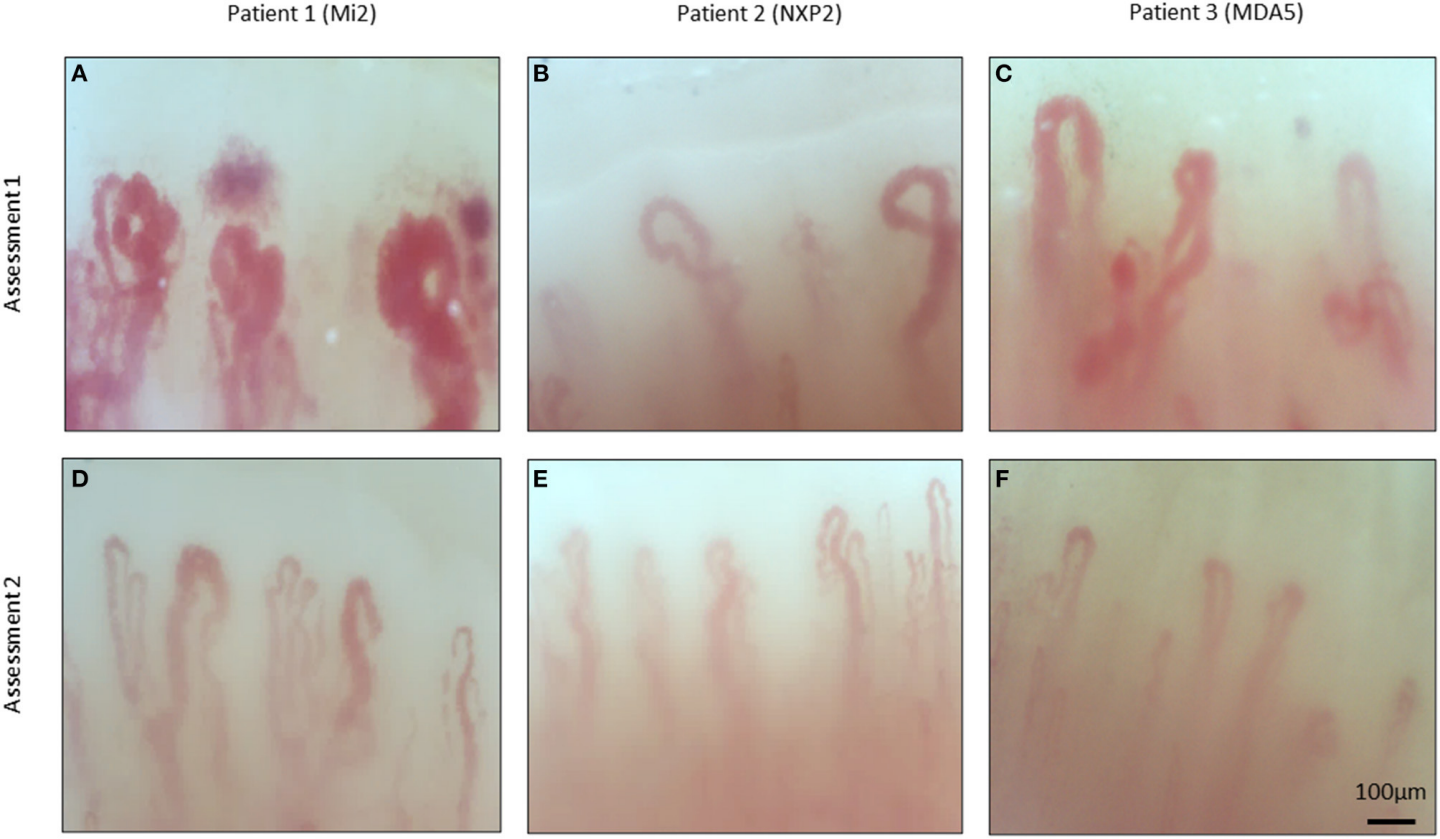
Nailfold video capillaroscopy images of three patients with dermatomyositis. Representative images of three patients with dermatomyositis taken during serial assessments. Myositis specific antibodies indicated in brackets. Abnormal features include presence of dilated capillaries **(A–D)**, microhemorrhages **(A,C)**, decreased capillary density **(A–D)**, and do not demonstrate and gross abnormalities **(E,F)**.

NVC abnormalities were detected in most patients at baseline assessment and are summarized in Table 1 and visualized in Figure 1. Frequent abnormalities included both decreased capillary density (80%) and the presence of microhemorrhages (80%). Giant and dilated capillaries were also present with a mean apical density of 56.09 μm (normal <22 μm). In follow-up, fewer NVC abnormalities were observed. There was significant recovery of both capillary density (4.71 vs. 6.84, *p* = 0.006) and capillary dilatation (56.09 vs. 27.79, *p* = 0.003). Fewer microhemorrhages were also detected and approached statistical significance (0.65 vs. 0.29, *p* = 0.053).

### Correlates of Disease Activity

**Figure 2:**
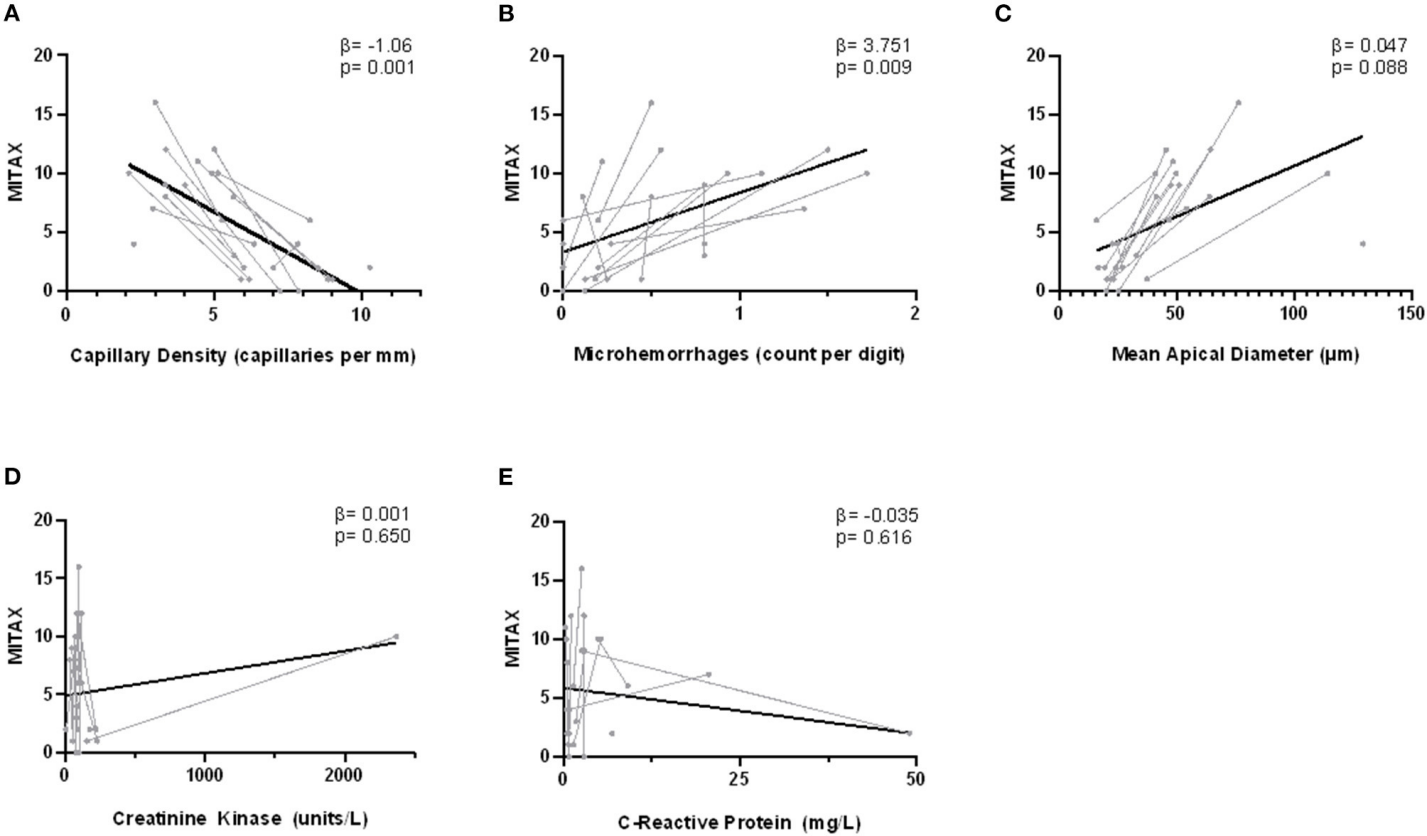
Correlation between nailfold capillary measurements and MITAX. Disease activity is indicated on the y-axis as measured by the Myositis intention to treat index (MITAX). The NVC measurements capillary density **(A)**, microhemorrhages **(B)**, and apical diameter **(C)** as well as biochemical indices creatinine kinase (CK) **(D)** and C-reactive protein (CRP) **(E)** are indicated on the x-axes. Single assessments are indicated in gray, with lines connecting the longitudinal assessments for one individual patient. The overall fitted line is indicated in black. Fixed effect estimates (β) and significance (*p*) are the result of individual mixed-linear models with dependent variable MITAX and time as a random effect.

Using a series of mixed-linear models, we analyzed the association between DM disease activity, as measured by MITAX, and various clinical parameters over the course of two consecutive assessments (Figure 2). No relationship was detected between MITAX and either CRP (*p* = 0.62) or CK (*p* = 0.65). In contrast, both decreasing capillary density and the presence of microhemorrhages were associated with increased disease activity. Those with more active disease also tended to have increased apical capillary diameters, although this did not achieve statistical significance.

To further characterize the relationship between NVC abnormalities and disease activity, we also performed a multiple mixed-linear model regression of NVC parameters and MITAX. Capillary density was independently associated with disease activity (β = −1.49 [CI −2.49, −0.33], *p* = 0.013), while apical diameter (β = −0.05 [CI −0.12, 0.02], *p* = 0.14), and microhemorrhages (β = 1.09 [CI −1.96, 4.13], *p* = 0.47) were not.

Finally, we examined the association between capillary density and individual MITAX components over time in a mixed-linear regression (Table 2). In additional to its association with global MITAX, there was a significant association between decreased capillary density and cutaneous, muscle, and constitutional disease. No relationship was detected for capillary density and GI, pulmonary, and skeletal disease activity, all of which were observed infrequently in our cohort.

**Table 2:** Association of capillary density measurements with individual and composite MITAX scores.

	**β**	***p***	**95% CI**
Global MITAX	−1.04	0.001	−1.61, −0.46
Cutaneous	−0.38	0.019	−0.69, −0.07
Muscle	−0.47	0.003	−0.76, −0.17
Constitutional	−0.20	0.006	−0.34, −0.06
Skeletal	0.08	0.099	−0.02, 0.18
GI	0.01	0.774	−0.08, 0.10
Pulmonary	0.01	0.867	−0.16, 0.19

*Fixed effect estimates (β), significance (p), and 95% confidence interval (CI) from mixed linear model regression of capillary density with MITAX (Myositis intention to treat index) components and time as a random effect*.

## Discussion

The presence of microvascular abnormalities, including microhemorrhages, giant capillaries, and capillary dropout have been well-described in DM (8). These abnormalities are dynamic and change over the course of disease. Patients with DM duration of <6 months had worse capillary drop out and giant capillaries than those with >6 months duration, suggesting that microvascular changes can resolve over time (9). Multiple studies have also demonstrated that the presence of microhemorrhages and decreased capillary density can improve over the course of treatment (10, 11). This is in keeping with our own findings of significant improvement in capillary density and dilatation in follow-up.

Given that small vessel vasculopathies leads to both clinical disease and nailfold capillary abnormalities in DM, it is reasonable to hypothesize that NVC and disease activity may be correlated. To date, there have been variable reports of the association between microvascular abnormalities and disease activity in DM (8). Several studies have reported an association between multiple NVC abnormalities and global DM disease activity (10, 12). By contrast, a cross sectional analysis of 50 DM patients found that NVC abnormalities were significantly associated with active muscle disease and only marginally with global disease activity (*p* = 0.56) (11).

Several studies, however, have not shown a relationship between NVC abnormalities and disease activity. One analysis found that the presence of NVC abnormalities correlated with EMG abnormalities, but not with disease activity in 27 patients with DM (13). Similarly, two cross-sectional studies found no relationship between NVC and disease activity (14, 15).

While small studies have shown variable results in adult DM, the relationship between nailfold capillary abnormalities and disease activities has been well-established in juvenile DM. A prospective analysis of 92 juvenile DM patients with repeated assessments over a period of 5.5 years demonstrated a strong correlation between the capillary density and both skin and muscle disease activity (16). This is corroborated by several other studies in juvenile DM that also report associations between NVC abnormalities and active cutaneous and/or muscle disease (17–19). These findings are congruent with analysis of our cohort, wherein decreased capillary density strongly correlated with disease activity over time. NVC assessment may therefore be useful not only for diagnosis, but for ongoing disease monitoring.

While our study and others have demonstrated that NVC findings correlate with cutaneous and muscle disease, they may also correlate with ILD in DM. The presence of multiple capillary abnormalities was found to be associated with a concurrent diagnosis of ILD (12). It has also been reported the presence of enlarged capillaries correlates with the presence of pulmonary involvement (15). Furthermore, the presence of microhemorrhages have also been correlated with ILD severity in a combined analysis of patients with MDA-5 antibody positive dermatomyositis and anti-synthetase syndrome (20). Ultimately, only two patients in our cohort had pulmonary involvement and we were likely underpowered to detect a relationship between ILD and NVC.

CK is a marker of muscle damage and is frequently measured during both diagnosis and follow-up of DM. However, in comparison to other inflammatory myopathies CK elevation in DM is relatively modest (21). CK abnormalities are also variable across DM subtypes, with only 41% of those with MDA-5 antibody positive disease having CK elevation, in comparison to 94.5% of those with MDA-5 antibody negative disease (22). In addition, CK elevation in DM is also known to be impacted by patient age, sex, and ethnicity (23).

While CK has been shown to have moderate correlation with muscle disease and is included by IMACS as a core measure of disease activity (24), it may not represent other manifestations and its utility as a biomarker of global disease activity in DM is unclear (1). In particular, for those patients that have resolution of muscle disease and more refractory cutaneous and pulmonary involvement, serial CK measurements may have limited utility in monitoring disease activity. During the creation of the 2016 ACR-EULAR DM response criteria, expert consensus deemed muscle enzymes evaluation to be less meaningful than physician global assessment, patient global assessment, HAQ, manual muscle testing, and assessment of extramuscular manifestations (25). As such, muscle enzyme evaluation has the smallest weighting of all core criteria in the 2016 ACR-EULAR adult DM response criteria, and no weighting in the juvenile DM criteria.

In our cohort, CK did not correlate with disease activity. All patients except one had a normal CK level, including those with active muscle disease. While CK has an established role in the diagnosis of DM, our data does not support its use as a sensitive biomarker of disease activity.

Few other biochemical markers are used clinically in the follow up of DM. While CRP is commonly tracked in DM patients, and has been correlated with the presence of ILD (26), it has not been found to correlate well with global disease activity (27). Similarly, our study found no correlation between overall disease activity and CRP elevation. The limited utility of readily available biochemical assays clearly defines the need for a reliable marker of disease activity.

### Limitations

This study is the first to prospectively demonstrate the relationship between longitudinal NVC findings and global disease activity in adult DM. Important limitations include its single-center design and small sample size. Additionally, we were likely underpowered to analyze the association between NVC findings and uncommon manifestations of DM such as pulmonary and GI disease.

## Conclusions

Microvascular changes are present in adult DM and are dynamic over time. Decreased capillary density strongly correlates with global disease activity as measured by MITAX and is more sensitive than traditional biochemical measures such as CK or CRP. Longitudinal NVC assessments may therefore represent an inexpensive and non-invasive measure of DM disease activity. Further studies with larger numbers of patients will be required to confirm these findings as well as understand the relationship between NVC and uncommon manifestations such as ILD.

## Data Availability Statement

The raw data supporting the conclusions of this article will be made available by the authors, without undue reservation.

## Ethics Statement

The studies involving human participants were reviewed and approved by University of Alberta Research Ethics Office. The patients/participants provided their written informed consent to participate in this study.

## Author Contributions

Study design was primarily performed by DJ and MO with contribution from all authors. NM, DR, CP, SK, RG, JC, and MO recruited and assessed study participants. DJ, CE, and MO performed data analysis. DJ and MO wrote the manuscript. All authors contributed to the article and approved the submitted version.

## Conflict of Interest

The authors declare that the research was conducted in the absence of any commercial or financial relationships that could be construed as a potential conflict of interest.

## Publisher's Note

All claims expressed in this article are solely those of the authors and do not necessarily represent those of their affiliated organizations, or those of the publisher, the editors and the reviewers. Any product that may be evaluated in this article, or claim that may be made by its manufacturer, is not guaranteed or endorsed by the publisher.
